# Emerging Biosensing Technologies for Neuroinflammatory and Neurodegenerative Disease Diagnostics

**DOI:** 10.3389/fnmol.2018.00164

**Published:** 2018-05-16

**Authors:** Catarina M. Abreu, Ricardo Soares-dos-Reis, Pedro N. Melo, João B. Relvas, Joana Guimarães, Maria José Sá, Andrea P. Cruz, Inês Mendes Pinto

**Affiliations:** ^1^International Iberian Nanotechnology Laboratory, Braga, Portugal; ^2^Medical School, Swansea University, Swansea, United Kingdom; ^3^Neurology Department, Centro Hospitalar de São João, Porto, Portugal; ^4^Department of Clinical Neurosciences and Mental Health, Faculdade de Medicina, Universidade do Porto, Porto, Portugal; ^5^Department of Biomedicine, Faculdade de Medicina, Universidade do Porto, Porto, Portugal; ^6^Graduate Programme in Areas of Basic and Applied Biology, Instituto de Ciências Biomédicas Abel Salazar, Universidade do Porto, Porto, Portugal; ^7^Instituto de Investigação e Inovação em Saúde, Universidade do Porto, Porto, Portugal; ^8^Center for Drug Discovery and Innovative Medicines (MedInUP), Universidade do Porto, Porto, Portugal; ^9^Energy, Environment and Health Research Unit (FP-ENAS), University Fernando Pessoa, Porto, Portugal; ^10^Faculty of Health Sciences, University Fernando Pessoa, Porto, Portugal

**Keywords:** neuroinflammation, biomarkers, Alzheimer's disease, Parkinson's disease, Multiple Sclerosis, biosensors, multiplex

## Abstract

Neuroinflammation plays a critical role in the onset and progression of many neurological disorders, including Multiple Sclerosis, Alzheimer's and Parkinson's diseases. In these clinical conditions the underlying neuroinflammatory processes are significantly heterogeneous. Nevertheless, a common link is the chronic activation of innate immune responses and imbalanced secretion of pro and anti-inflammatory mediators. In light of this, the discovery of robust biomarkers is crucial for screening, early diagnosis, and monitoring of neurological diseases. However, the difficulty to investigate biochemical processes directly in the central nervous system (CNS) is challenging. In recent years, biomarkers of CNS inflammatory responses have been identified in different body fluids, such as blood, cerebrospinal fluid, and tears. In addition, progress in micro and nanotechnology has enabled the development of biosensing platforms capable of detecting in real-time, multiple biomarkers in clinically relevant samples. Biosensing technologies are approaching maturity where they will become deployed in community settings, at which point screening programs and personalized medicine will become a reality. In this multidisciplinary review, our goal is to highlight both clinical and recent technological advances toward the development of multiplex-based solutions for effective neuroinflammatory and neurodegenerative disease diagnostics and monitoring.

## Neurodegeneration and inflammation: a clinical and molecular perspective

Neurological disorders account for an increasing number of disability-adjusted life-years worldwide, especially in high-income countries. Alzheimer's disease, Parkinson's disease and Multiple Sclerosis are the most prevalent causes of neurological disability (Hay et al., 2017). The three different conditions share features of neurodegeneration and neuroinflammation and their diagnosis rely mainly on clinical examination, complemented by imaging and biomarker analysis (Table 1) (Poewe et al., 2017; Lane et al., 2018; Reich et al., 2018).

**Table 1:** Biomarkers of neurodegeneration and neuroinflammation in Multiple Sclerosis, Parkinson, and Alzheimer's disease^a^.

	**Disorder**	**Biomarker type**	**Current biomarkers**	**Inflammatory biomarkers**	**Neurodegeneration biomarkers**	**AUC**
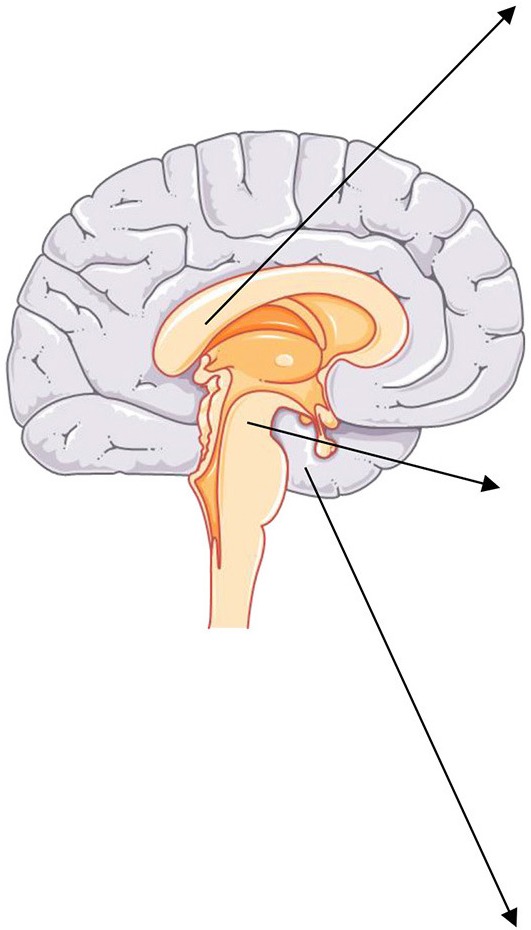	**MS**	Clinical	Neurological disability	Relapse	EDSS progression	–
		Imaging	MRI w/gadolinium	^11^C-PK11195 PET (mainly plaques) (Inglese and Petracca, 2013)	Brain atrophy (MRI); ^11^C-flumazenil PET (Inglese and Petracca, 2013)	–
		Serum	–	TNFα, IL-1β, RANKL, IL-17, PTX3, IL-10 (D'Ambrosio et al., 2015); OPN (Housley et al., 2015)	Nfl (Zetterberg, 2017); Nfh (Housley et al., 2015)	Nfl (0.663) (Novakova et al., 2017b); IFNγ (0.91) (Arellano et al., 2017)
		CSF	Oligoclonal bands; IgG index	CHI3L1 (Novakova et al., 2017a); CXCL13, IL-23, IL-17, CXCL10, TNFα, TGF-β (Kothur et al., 2016); CHIT1, MCP-1, GFAP (Novakova et al., 2017a); sTREM2 (Zetterberg, 2017); OPN (Housley et al., 2015)	Nfl, NGRN (Novakova et al., 2017a); Nfh (Housley et al., 2015); NAA(low) (Teunissen et al., 2015)	Nfl (0.774) (Novakova et al., 2017b); CHI3L1 (0.82) (Opsahl et al., 2016); CXCL13 (0.80) (Stilund et al., 2015)
	**PD**	Clinical	Bradykinesia; Rigidity; Resting tremor	–	Disability	–
		Imaging	MIBG scintigraphy; SPECT (^123^I-ioflupane)	^11^C-PK11195 PET (midbrain/putamen) (Inglese and Petracca, 2013)	–	–
		Serum	–	IFNγ, IL-1β, IL-2, IL-3, IL-10, MIF, TNFα (Rocha et al., 2015); α-syn specific T-cells (Sulzer et al., 2017); N-glycated IgG (Russell et al., 2017) MIP-1β, MCP-1, IL-8 (Brockmann et al., 2017)	–	Nfl (0.91) (Hansson et al., 2017); IL-8 (0.895), MCP-1 (0.736); MIP-1β (0.767) (Brockmann et al., 2017); N-glycated IgG (0.92) (Russell et al., 2017)
		CSF	–	β2-microglobulin, IL-8, IL-6, TNFα, CHI3L1 (Andersen et al., 2017)	Dopamine (loss), Nfl (low) (Andersen et al., 2017)	TNFα (0.658) (Delgado-Alvarado et al., 2017)
	**AD**	Clinical	Progressive episodic memory loss	–	Loss of autonomy	–
		Imaging	PET-PiB; MRI	^11^C-PK11195 PET (temporo-parietal cortex) (Inglese and Petracca, 2013)	MRI; ^11^C-flumazenil PET (Pascual et al., 2012)	–
		Serum	-	CHI3L1 (Olsson et al., 2016); IL-8 (Popp et al., 2017); FGF-1, IL-1β, IL-10, IL-11, IL-18 (Brosseron et al., 2014), IL-3, MCP-1, RANTES, sIL-6R, TGF-β1 (Delaby et al., 2015); sCD40L (Yu et al., 2016)	Total tau (Olsson et al., 2016); NSE (Huynh and Mohan, 2017)	IL-8 (0.599), IL-3 (0.549), MCP-1 (0.501), RANTES (0.556), sIL6-R (0.595), TGF-β1 (0.567) (Delaby et al., 2015); sCD40L (0.824) (Yu et al., 2016)
		CSF	Aβ_1−42_; total tau; p-tau	CHI3L1, MCP-1 (Olsson et al., 2016); IL-15, sFLT-1, sICAM-1 (Popp et al., 2017), MIP-1β, MIP-3β, sIL-6R (Delaby et al., 2015); IL-1β (Hesse et al., 2016)	VILIP-1 (Huynh and Mohan, 2017); NGRN (Novakova et al., 2017a); Nfl, NSE, HFABP (Olsson et al., 2016);	MCP-1 (0.503), MIP-1β (0.655), MIP-3β (0.727). p-tau (0.946), sIL-6R (0.755), tau (0.942) (Delaby et al., 2015); CHI3L1 (0.75) (Paterson et al., 2016); IL-8 (0.614) (Delaby et al., 2015); IL-1β (0.62) (Hesse et al., 2016)

a *The table above is not intended as an exhaustive review. Only markers studied in human subjects were included. No animal or ex-vivo data was considered. Only one PET ligand per molecule was considered (e.g., other ligands for TSPO or amyloid exist). AUC of inflammatory biomarkers indicated when available (values are highly cohort specific and vary according to disease, test sample and quantification method). AD, Alzheimer's Disease; AUC, Area Under Curve; ^11^C-PK11195, a TSPO radioligand reflecting microglial activation; CHI3L1, chitinase-3-like protein 1; CHIT1, chitotriosidase; CXCL13, C-X-C motif ligand 13; EDSS, Kurtzke Expanded Disability Status Scale; FGF, fibroblast growth factor; GFAP, glial fibrillary acidic protein; HFABP, Heart fatty acid binding protein; MCP-1, monocyte chemoattractant protein-1; MIBG, metaiodobenzylguanidine; MIP, Macrophage Inflammatory Proteins; MRI, magnetic resonance imaging; MS, Multiple Sclerosis; NAA, N-acetylaspartate; Nfl, neurofilament light chain protein; Nfh, neurofilament heavy chain protein; NGRN, neurogranin; NSE, neuron-specific enolase; OPN, osteopontin; PD, Parkinson's Disease; PET, Positron Emission Tomography; PiB, Pittsburgh compound B; PTX3, pentraxin 3; RANKL, receptor activator of nuclear factor kappa-B ligand; SPECT, Single-photon emission computed tomography; sFLT-1, soluble fms-related tyrosine kinase 1; sICAM-1, soluble intercellular adhesion molecule 1; sIL-6R, Soluble Interleukin-6 receptor; sTREM2, secreted form of the triggering receptor expressed on myeloid cells 2; TGF-β1, transforming growth factor beta 1; VILIP-1, Visinin-like protein 1*.

Alzheimer's disease (AD) is a neurodegenerative disorder primarily affecting neocortical regions and characterized by progressive episodic memory loss leading to significant behavioral changes (McDonald et al., 2009; Lane et al., 2018). Definite AD diagnosis is histopathological, while diagnosis of probable/possible AD dementia is only made by clinical assessment. Diagnostic accuracy can be enhanced by further findings of low amyloid-beta (Aβ) levels and an increase in the total or phosphorylated tau protein in cerebrospinal fluid (CSF) (McKhann et al., 2011). Furthermore, positron emitting tomography (PET) showing increased amyloid deposition or decreased fluorodeoxyglucose uptake in the temporo-parietal cortex also represent acceptable evidence of the AD pathophysiological process (McKhann et al., 2011). Overall, AD pathology has been classically associated to the presence of amyloid plaques (neuritic plaques) and hyperphosphorylated tau aggregates (neurofibrillary tangles, NFTs) in the brain, which titrates the corresponding levels in the CSF. Amyloid plaques are believed to arise from an imbalance between Aβ_1−42_ production [via γ and β-secretase 1 (BACE1) cleavage of amyloid precursor protein] and its clearance, leading to the formation of toxic oligomers (AβO), subsequent synaptic dysfunction and neuronal cell death (Lane et al., 2018). In dominant inherited forms of AD (including mutations in γ-secretase subunits, *PSEN*1 and *PSEN2)* the formation of amyloid plaques is promoted by an increased production of Aβ_1−42_, while in sporadic AD it is mainly due to impaired Aβ clearance (Mawuenyega et al., 2010; Lane et al., 2018). Mutations in genes coding for proteins involved in Aβ clearance pathways represent risk factors for AD, among these are apolipoprotein E (APOE) and the immune receptors: triggering receptor expressed on myeloid cells 2 (TREM2), cluster of differentiation 33 (CD33), and complement region 1 (CR1). TREM2, CD33, and CR1 are expressed in microglia, the innate immune cells of the central nervous system (CNS) and have been found to be associated with a higher risk of AD (Polvikoski et al., 1995; Bradshaw et al., 2013; Crehan et al., 2013; Griciuc et al., 2013; Guerreiro et al., 2013; Farfel et al., 2016). Microglia activation can have a neurotoxic role in AD through activation of the complement system (e.g., C1q, C3) and the inflammasome, release of pro-inflammatory mediators [e.g., interleukin-1 (IL-1), IL-6 and tumor necrosis factor α (TNFα)] and leading to synaptic loss, mitogen-activated protein kinase (MAPK) activation and subsequent NFTs formation (Griffin et al., 2006; Heneka et al., 2013; Dursun et al., 2015; Wang et al., 2015; Hong et al., 2016; Fonseca et al., 2017; Liddelow et al., 2017). Despite the supporting evidence of the innate immunity pathways in AD pathogenesis, attempts to modulate the inflammatory response in patients with AD have mostly failed at improving cognition and halting disease progression (Bronzuoli et al., 2016; Dansokho and Heneka, 2017; Honig et al., 2018).

Parkinson's disease (PD), the second most common neurodegenerative disorder, is characterized by the early and progressive loss of dopaminergic neurons in the *substantia nigra pars compacta* associated with abnormal α-synuclein (α-syn) deposition (Kalia and Lang, 2015). The resulting striatal dopamine deficiency leads to a movement disorder with a clinically recognizable triad of motor symptoms: bradykinesia (“slow movement”) together with resting tremor and/or rigidity initially restricted to one limb or hemibody, slowly progressing to affect the rest of the body. However, PD is also associated with pathological changes in other brain regions causing non-motor symptoms (e.g., hyposmia, dysautonomia, sleep, and psychiatric/cognitive disorders) that add to overall disability and can precede motor dysfunction (Kalia and Lang, 2015). These likely reflects the distribution of α-syn aggregates to other regions of the nervous system (Postuma et al., 2015; Poewe et al., 2017). PD diagnosis is exclusively clinical. However, ancillary tests include metaiodobenzylguanidine (MIBG) scintigraphy demonstrating cardiac sympathetic denervation, olfactory function testing and pre-synaptic dopamine (DA) receptor ^123^I-ioflupane single-photon emission computed tomography (SPECT) imaging. Biomarker analysis, including α-syn, in serum or CSF, is not performed in standard clinical practice (Postuma et al., 2015). Nevertheless, α-syn aggregates in specific brain regions are recognized neuropathological hallmarks of PD. In fact, α-syn mutation is responsible for heritable forms of PD (Poewe et al., 2017). Other genes identified in inherited PD and corresponding proteins, include *PARK7* (deglycase DJ-1), *GBA* (glucocerebrosidase), *PRKN* (parkin), and *LRRK2* (leucine-rich repeat kinase 2) which are expressed in microglia (Lee et al., 2017). At large, the physiological functions of PD-associated genes in immune cells remain elusive. Nevertheless, it is possible that mutations in those genes can alter their normal microglia functions worsening the progression of inflammation-mediated PD neurodegeneration (Lee et al., 2017). Studies found signs of microglia activation and chronic inflammation in the brains of PD patients (McGeer et al., 1988; Gerhard et al., 2006) and α-syn aggregates are capable of activating microglia *in vitro* and in mouse models (Brochard et al., 2009). Pro-inflammatory cytokines, such as TNFα, IL-1β, IL-6, IL-2, and IL-10 are increased in *postmortem* brain (Mogi et al., 1994b), CSF (Mogi et al., 1994a), and serum (Dufek et al., 2009; Williams-Gray et al., 2016) of PD patients and may be predictive of disease progression.

Multiple Sclerosis (MS) is a chronic inflammatory demyelinating disorder of the CNS of unknown etiology but with a genetic predisposition and environmental influence (Dendrou et al., 2015; Reich et al., 2018). Initial symptoms are variable and related with the affected area of the CNS (Mowry et al., 2009; Pires et al., 2016). Diagnosis of MS requires clinical or radiological evidence of lesion dissemination in time and/or space. Magnetic resonance imaging (MRI) is the conventional diagnostic tool, while serum and CSF testing are useful in excluding other pathologies. The presence of CSF-restricted oligoclonal bands (OCBs) supports MS diagnosis, however it is not MS-specific (Thompson et al., 2017). CSF-restricted OCBs can be found in other diseases whose clinical and imaging characteristics differ from MS, such as systemic inflammatory disorders with CNS expression (e.g., Systemic Lupus Erythematous, Sarcoidosis, and Behçet's disease), CNS infections (e.g., Neurosyphilis, HIV, Neuroborreliosis, Subacute Sclerosing Panencephalitis) and in some hereditary disorders (e.g., Ataxia-telangiectasia and Adrenoleukodystrophy) (Giovannoni, 2014).

In MS lesions, histopathology reveals profound myelin loss, increased inflammatory response, and secondary axonal degeneration. Microglia activation perpetuates the underlying inflammatory response at the demyelinated plaque and at sites remote from the lesion (Dendrou et al., 2015). Microglia-driven production of reactive oxygen and nitrogen species, which stress the neuronal and mitochondrial metabolism, promotes neuronal death (Schuh et al., 2014; Choi et al., 2017; Luo et al., 2017) which leads to the release of cytoskeletal elements into the CSF, such as neurofilaments (NFs). NFs are promising biomarkers for predicting lesion burden, therapeutic response, and disease progression (Zetterberg, 2017).

CNS tissue damage in MS results from an intricate interplay between the immune system, glial cells, and neurons. Although there is ongoing debate regarding MS origin, i.e., the “outside-in” (peripheral immune cell invasion of the CNS) or “inside-out” (CNS-intrinsic initiation of the inflammatory cascade) models (Reich et al., 2018), studies in animal models, and in patient CSF and blood samples have disclosed a critical role for adaptive immunity (auto-reactive T and B cells and autoantibodies) (Reich et al., 2018). Despite the knowledge gap regarding MS initial immunopathogenesis, therapies directed both at T cells and B cells have been effective in reducing relapse rate and disease progression (Pires et al., 2016; Reich et al., 2018).

Although the trigger for inflammation might be specific for each of the diseases mentioned above, evidence suggests that AD, PD, and MS share common cellular and molecular mechanisms for sensing, transducing and amplifying inflammation that results in the production of mediators of inflammation, neurotoxicity and, ultimately, neuronal cell death (Yadav et al., 2015; Guillot-Sestier and Town, 2017). Activation of microglial cells is a key event in such neuroinflammatory processes (Ginhoux and Guilliams, 2016). Under physiological conditions microglia assume immune surveillance functions but upon tissue damage or infection they change their morphology and transcriptomic profile enabling them to restore tissue homeostasis (Crotti and Ransohoff, 2016). Through pattern recognition receptors, including TREM2, microglial cells recognize environmental cues that instruct them to initiate inflammatory responses by triggering downstream signaling pathways regulating the activity of the transcription factors AP-1 and NF-κB, which in turn control the production and release of inflammatory mediators, such as the cytokines TNFα, IL-1β, IL-6, and IL-8, reactive oxygen and nitrogen species (Ortiz et al., 2013; Leszek et al., 2016; Labzin et al., 2018). The analysis of inflammatory profile, in association with classical disease-specific biomarkers could potentially increase diagnostic and prognostic accuracy (Table 1).

## Sensing circulating biomarkers of neurodegeneration and neuroinflammation

Over the past years, great efforts have been made to identify biomarkers associated with CNS diseases in clinically relevant samples. A biomarker is defined as a measurable biologically plausible parameter, usually being an indicator of an underlying disease mechanism (Atkinson et al., 2001). In addition, an ideal biomarker should also be readily accessible, highly sensitive, and specific and its levels should correlate with disease progression and/or treatment response, allowing disease risk stratification (Bennett and Devarajan, 2016). Biomarker cut-off values determine the clinical sensitivity (ratio of true positives over all individuals with disease) and specificity (ratio of true negatives over all individuals without disease). The Receiver Operating Characteristic (ROC) curve is a graphic display of sensitivity versus (1-specificity), and its Area Under the Curve (AUC) provides a useful measure for optimal cut-off value selection (Parikh and Thiessen-Philbrook, 2014). AUC values for single biomarkers are shown in Table 1. Recent studies suggest that the combination of multiple biomarkers increases the AUC value, therefore increasing the accuracy of the disease diagnostic tests (Spellman et al., 2015; Lue et al., 2016).

Although many neurological studies have relied on the biochemical analysis of CSF, the physiological sample of reference for CNS disorders, these biomarkers are also present in more accessible biological fluids, making sample acquisition less invasive, as exemplified for TNFα and OCBs that are present in higher amounts in tears of PD and MS patients, respectively (Devos et al., 2001; Çomoglu et al., 2013). Nevertheless, this biochemical profiling has mostly relied on microarray technologies (Choi et al., 2008; Craig-Schapiro et al., 2011; Martins et al., 2011; Koziorowski et al., 2012; Edwards et al., 2013; Laske et al., 2013; Burman et al., 2014; Delaby et al., 2015; Cala et al., 2016; Hegen et al., 2016; Lue et al., 2016) and liquid chromatography-mass spectroscopy (Musunuri et al., 2014; Hölttä et al., 2015; Spellman et al., 2015; Paterson et al., 2016) which, although effective for large biomarker panel assessment, are not suitable for point-of-care testing. On the other hand, identification and validation of potential biomarkers is often hindered by their low concentrations in the test fluid and inherent variability across control and patient samples. As such, there is a need for new technologies with lower limit of detection (LOD) and higher sensitivity.

Biosensors are analytical devices capable of converting specific biorecognition events into a measurable signal. Conventional biosensors are composed of a receptor (e.g., antibody, enzyme, and DNA) which specifically recognizes the biomarker (e.g., antigen, enzyme substrate, and DNA) of interest and a transducer which converts biochemical interactions into a quantifiable electrical signal proportional to biomarker concentrations. Biosensors are commonly classified in electrochemical, optical, piezoelectric, or magnetic, based on the signal transduction mechanism. These technologies have broad applications in health (Zhang et al., 2017; El Harrad et al., 2018), food (Law et al., 2014; Vasilescu and Marty, 2016), and environmental sciences (Rapini and Marrazza, 2017; Kumar et al., 2018). Over the past years, the critical role of inflammation in disease has led researchers to develop biosensors for the specific detection of inflammatory mediators in clinically relevant body fluids. Although most inflammation-targeted biosensors have not been tested in the context of neuroinflammatory diseases, the clinical potential of these technologies is undeniable (Table 2). Recently, Baraket et al. developed an electrochemical biosensor to monitor IL-1β and IL-10 cytokine levels after the implantation of left ventricular assist devices (LVADs) in patients with heart failure while waiting for compatible donors (Baraket et al., 2017). Given the non-biocompatible nature of the LVAD, many patients suffer from acute inflammation in which several pro and anti-inflammatory cytokines are secreted, such as IL-1β and IL-10, respectively. The proposed biosensor was capable of detecting both cytokines within the range of 1–15 pg/mL, relevant to predict the first signs of inflammation (Stumpf et al., 2003).

**Table 2:** Biosensing technologies for neurodegenerative disease diagnostics and monitoring.

**Disease**	**Biomarker**	**Application**	**Transduction platform**	**Sample**	**LOD**	**Detection time**	**References**
Inflammation	IL-1β	Patient monitoring	Optical	Patient Serum	158.5 fg/mL (PBS) 1 pg/mL (diluted serum)	<15 min (total)	Song et al., 2017
	IL-1β and IL-10	Patient monitoring	Electrochemical	Spiked in buffer	0.3 pg/mL (IL-10) 0.7 pg/mL (IL-1β)	45 min (total)	Baraket et al., 2017
	IL-10	Patient monitoring	Electrochemical	Spiked in buffer	–	30 min (incubation)	Baraket et al., 2016
	IL-6	Drug screening	Electrochemical	Nasopharyngeal carcinoma cell line	–	48 h (total)	Lei et al., 2016
	IL-6	Patient monitoring	Electrical	Spiked in buffer	1.53 pg/mL	Real-time	Huang et al., 2015
	TNFα	Patient monitoring	Electrochemical	Spiked Serum	60 pg/mL	20 min (incubation)	Arya and Estrela, 2017
	TNFα	Patient monitoring	Electrochemical	Spiked Serum and Saliva	3.7 fg/mL	45 min (incubation)	Aydin et al., 2017
	IL-12	Diagnosis	Electrochemical	Spiked in FBS	3.5 pg/mL	20 min (incubation)	Bhavsar et al., 2009
	MMP-9	Patient monitoring	Electrochemical	Spiked in buffer	15 ng/mL	-	Biela et al., 2015
	IFNγ	Patient monitoring	Electrochemical	Spiked Serum	0.048 pg/mL	35 min (incubation)	Zhang et al., 2016
	IL-2, IL-4, IL-6, IL-10, TNFα, IFNγ	Patient monitoring	Optical	Patient Serum	20.56 pg/mL (IL-2) 4.60 pg/mL (IL-4) 11.29 pg/mL (IL-6) 10.97 pg/mL (IL-10) 11.43 pg/mL (TNFα) 6.46 pg/mL (IFNγ)	40 min (total)	Chen et al., 2015
AD	Aβ_1−42_ peptide	Diagnosis Patient monitoring	Electrochemical	Spiked in buffer	5.2 pg/mL	10 min (incubation)	Carneiro et al., 2017
			Immunomagnetic	Spiked in artificial CSF	5.0 pg/mL	30 min (incubation)	Li et al., 2016
			Electrical	Spiked in buffer and plasma of mice	0.1 pg/mL	20 min (incubation)	Kim et al., 2016a
				Spiked in serum	1.0 pg/mL	Real-time	Oh et al., 2013
	Aβ_1−42_ and total Aβ peptides	Diagnosis	Electrochemical	Spiked in artificial CSF	5 pM	80 min (total)	Liu et al., 2014
	Aβ_1−42_ and Aβ_1−40_ peptides	Diagnosis Fundamental studies	Electrochemical (Multiplex)	Spiked in CSF of mice	20 nM	~10 min (incubation)	Prabhulkar et al., 2012
			Optical (Multiplex with microfluidics)	Patient CSF	3.3 pM (Aβ_1−40_) 3.5 pM (Aβ_1−42_)	-	Xia et al., 2010
	Aβ_1−42_, Aβ_1−40_ peptides and tau protein	Diagnosis	Optical	Spiked in artificial plasma	34.9 fM (Aβ_1−40_) 26 fM (Aβ_1−42_) 23.6 fM (tau protein)	60 min (incubation)	Kim et al., 2018
	Aβ oligomer	Diagnosis Patient monitoring	Electrochemical	Conditioned media of 7PA2 CHO cells	0.5 pM	20 min (incubation)	Rushworth et al., 2014
				Spiked in artificial CSF	100 pM	60 min (incubation)	Zhou et al., 2016
				Spiked in Serum and CSF	6 pM	20 min (incubation)	Xing et al., 2017
			Optical	Spiked in buffer	0.2 nM	5 min (incubation)	Xia et al., 2016
	O-GlcNAc transferase activity	Drug screening Fundamental studies	Electrochemical	Spiked in buffer	–	~120 min (total)	Yang et al., 2017
	Tau protein	Diagnosis	Electrochemical	Spiked in serum	0.03 pM	25 min (incubation)	Wang et al., 2017
					1000 pg/mL	3 h (incubation)	Dai et al., 2017
	Acetylcholine	Diagnosis	Electrochemical	Spiked in serum	4 nM	4 s (total)	Chauhan et al., 2017
				Spiked in buffers	10 μM	3 min (total)	Moreira et al., 2017
				Serum	5 nM	3 s (total)	Chauhan and Pundir, 2014
	Apolipoprotein E	Diagnosis	Electrochemical	Spiked in buffer	286 nM	2h (incubation)	Cheng et al., 2014
			Optical	Spiked in buffer	5 μg/mL	15 min (total)	Sciacca et al., 2013
	Fibrinogen	Diagnosis	Optical	Patient plasma	20 ng/mL	2h (incubation)	Kim et al., 2016b
	BACE1	Diagnosis Patient Monitoring	Optical	Spiked plasma and cell lysates	500 fM	60 min (incubation)	Vilela et al., 2017
		Drug screening	Optical	BACE1 inhibitors	–	–	Christopeit et al., 2011
PD	Dopamine	Diagnosis	Optical	Spiked in buffer	40 nM	30 min (incubation)	Yildirim and Bayindir, 2014
			Electrical	Spiked samples	10 pM (PBS) 1 nM (Serum)	Real time (total)	Park et al., 2014
			Electrical	Spiked in buffer	100 fM	Real time (total)	Lee et al., 2015
			Optical	Spiked in CSF	0.830 nM	5 min (incubation)	Govindaraju et al., 2017
	Dopamine and Uric acid	Diagnosis	Electrochemical	Patient Serum	1 nM	–	Yue et al., 2014
	α-synuclein	Diagnosis	Photoelectrochemical	Spiked in buffer	34 pg/mL	60 min (incubation)	An et al., 2010
	Thrombin	Diagnosis	Electrochemical	Patient blood and CSF	1 fM	3h (total)	Heydari-Bafrooei et al., 2016
	Acetylcholinesterase	Drug screening	Photoelectrochemical	(R)-Sal; (R)-NMSal	–	–	Huang et al., 2013
MS	Autoantibodies	Diagnosis Patient monitoring	Optical	Patient serum	–	4 min (incubation)	Real-Fernández et al., 2012
			Electrochemical	Patient serum and CSF	0.1495 ng/mL (gelatin-TiO_2_-MBP)	30 min (incubation) (gelatin-TiO_2_-MBP)	Derkus et al., 2013
	Myelin Basic Protein Tau protein	Diagnosis	Electrochemical	Spiked serum and CSF	0.30 nM (Myelin basic protein) 0.15 nM (Tau protein)	–	Derkus et al., 2017

*LOD, Limit of detection; Aβ, Amyloid; (R)-Sal: 1(R)- methyl-6,7-dihydroxy-1,2,3,4-tetrahydroisoquinoline; (R)-NMSal: 1(R),2(N)-dimethyl-6,7-dihydroxy- 1,2,3,4-tetra-hydroisoquinoline*.

Increased levels of pro-inflammatory cytokines in the CSF and serum of MS patients can alter the permeability of the blood-brain-barrier and promote T-lymphocyte migration into the brain and disease progression (Khaibullin et al., 2017). Therefore, cytokine detection in minimally invasive body fluids represents an attractive alternative for timely diagnosis of MS patients. Moreover, it allows early identification of relapsing patients and prediction of anti-inflammatory therapy failure, of outmost interest for effective clinical intervention. Elevated serum levels of matrix metalloproteinase-9 (MMP-9) have been associated with ongoing neuroinflammation processes and are indicative of MS relapse (Fainardi et al., 2006). Biela et al. developed an electrochemical biosensor for the sensitive and rapid detection of MMP-9 in clinically relevant ranges (50–400 ng/mL) (Biela et al., 2015). The biosensor was coated with a hydrogel and cross-linked peptides with specific MMP-9 cleavage sites. Exposure to MMP-9 resulted in the degradation of the hydrogel-peptide film and, consequently, produced an electrochemical signal. Importantly, the authors confirmed the specificity of the biosensor for MMP-9 detection against MMP-2, also present in the blood. Additionally, an electrochemical biosensor for IL-12 detection was developed by Bhavsar et al. for automated real-time biomarker analysis (Bhavsar et al., 2009). Although the biosensor was not validated with patient samples, the authors confirmed IL-12 detection in spiked samples of fetal bovine serum, showing a LOD of 3.5 pg/mL, lower than reported values for IL-12 expression in MS patients (Drulović et al., 1998).

In 2015, Chen and co-workers introduced for the first time a biosensor for simultaneous detection of multiple cytokines (Chen et al., 2015) and real-time monitoring of the inflammatory response of two neonates after a cardiopulmonary bypass surgery. This technology is based on a microfluidic surface plasmon resonance (LSPR) sensor capable of detecting multiple analytes through refractometric measurements. The authors demonstrated parallel multiplex analysis of six cytokines (IL-2, IL-4, IL-6, IL-10, TNFα, interferon γ (IFNγ) with a linear range of detection between 5 and 20 pg/mL, only requiring 1 μL of serum sample. Conventionally, nanoplasmonic biosensors are not suitable for point-of-care medical applications due to their limited sensitivity and optical microscope requirements. Nevertheless, the authors employed dark-field imaging with nanorods conjugated with antibodies to improve the sensitivity 10 times more than conventional LSPR chips.

The quantification of inflammatory mediators in minimally invasive samples of patients with neurodegenerative diseases provides valuable clinical information regarding their immune status. Nevertheless, it is insufficient to provide an accurate diagnosis. A comprehensive analysis and quantification of disease-specific biomarkers allied with immune system surveillance may improve patient prognosis by allowing timely and accurate diagnosis while enabling patient stratification for personalized treatment (Table 2).

AD has been by far the most intensely studied neurodegenerative pathology toward the development of effective and sensitive diagnostic platforms with sensors targeting Aβ peptides and oligomers in blood and CSF (Oh et al., 2013; Kim et al., 2016a; Li et al., 2016; Carneiro et al., 2017). Of these, Carneiro et al. recently reported an electrochemical biosensor for the detection of Aβ_1−42_ with a LOD of 5.2 pg/mL and wide dynamic range (10–1,000 pg/mL) provided by the use of gold nanoparticles (NPs) (Carneiro et al., 2017). This is particularly significant for the assessment of Aβ_1−42_ levels which are below 500 pg/mL in CSF of AD patients (Gagni et al., 2013). Also, Rushworth et al. developed a novel, label-free impedimetric biosensor for the specific detection of AβO. A fragment of the cellular prion protein (PrP^C^ residues 95–110), which mediates the neuronal binding and toxicity of AβO, was used as a recognition element for the specific detection of the oligomers. The biosensor presented a LOD of 0.5 pM and successfully detected cell-derived AβO from conditioned media of 7PA2 Chinese Hamster Ovary (CHO) cells that naturally secrete AβO (Rushworth et al., 2014). Interestingly, to validate the detection of AβO in conditioned media, the authors cultured the cells in the presence of βIV (BACE1 inhibitor), which prevents the generation of AβO by inactivation of BACE1. This experiment clearly demonstrated the biosensor's capability of functioning as a reliable source of AβO detection for AD diagnosis while also validating its use as a drug screening platform for BACE1. In 2011, Christopeit and colleagues developed a sophisticated drug screening platform with immobilized BACE1 on a plasma membrane-mimicking lipid layer (Christopeit et al., 2011). Vilela et al. reported an optical biosensor based on graphene oxide and upconversion NPs for the specific detection of *BACE1* mRNA with a LOD of 500 fM (Vilela et al., 2017). The biosensor showed high specificity for *BACE1* detection in spiked samples of healthy patient's plasma and cell lysates as well as long-term storage stability, demonstrating the clinical potential of the sensor.

Although Aβ_1−42_ and tau protein are well-established as AD diagnostic markers, they fail to provide the necessary specificity for effective diagnosis and disease progression assessment. Recent evidence suggests that the combination of multiple biomarkers may provide a more reliable and accurate diagnosis. For instance, Lewczuk et al. verified that Aβ_1−42_/Aβ_1−40_ concentration ratio is a better predictor of AD than Aβ_1−42_ alone (Lewczuk et al., 2015). Given the preponderant role of neuroinflammation in AD, monitoring circulating inflammatory mediators, such as cytokines, chemokines, and growth factors could provide valuable insights for early screening and treatment response evaluation (Laske et al., 2013; Delaby et al., 2015). Nevertheless, multiplex biosensor development for AD is still scarce, with only a few studies focused on Aβ detection (Xia et al., 2010; Liu et al., 2014).

Currently available biosensors are targeting markers such as the acetylcholine neurotransmitter (Chauhan and Pundir, 2014; Chauhan et al., 2017; Moreira et al., 2017), which is essential for memory processing, fibrinogen (Kim et al., 2016b), a clotting protein associated with Aβ aggregation (Cortes-Canteli et al., 2012) and APOE (Sciacca et al., 2013; Cheng et al., 2014). Nevertheless, single biomarker detection has fallen short for reliable AD diagnosis. Recently, Yang et al. devised an electrochemical biosensor for small-molecule O-GlcNAc transferase (OGT) inhibitor screening as an alternative to the conventional approaches (Yang et al., 2017). As it is known that aberrant activity of OGT may be involved in neurodegeneration and AD (Yuzwa and Vocadlo, 2014), the screening of OGT inhibitors could potentially lead to the development of targeted therapeutics and protein glycosylation pathway research. In this work, the authors studied the impact of concentration and incubation time of benzoxazolinone (BZX) and alloxan, which are known OGT inhibitors. This proof-of-concept study paves the way for the optimization of a label-free integrated platform for high-throughput drug screening of OGT inhibitors, specifically if multiple analytes or enzymes for O-linked glycosylation are analyzed simultaneously.

For PD, Yildirim et al. reported an optical technique for the detection of dopamine (DA) based on its oxidation and subsequent aggregation into NPs (polydopamine) (Yildirim and Bayindir, 2014). Interestingly, these NPs hold fluorescent properties, which allow the determination of DA concentrations with a detection limit of 40 nM. Additionally, Yue et al. reported the development of an electrochemical biosensor of vertically aligned ZnO nanowires on a 3D graphene foam for the detection of DA, uric acid (UA), and ascorbic acid (AA) (Yue et al., 2014). The use of 3D graphene foam enhanced electron transport due to its high conductivity and the vertical ZnO nanowires provided higher surface area. Importantly, the authors demonstrated the selectivity of the assay for DA, UA, and AA detection. The development of electrochemical biosensors for the specific detection of these molecules is particularly challenging, as they co-exist in serum with similar redox potential, thus limiting their oxidative peak discrimination. Of note, they verified that the UA serum levels for healthy individuals ranged from 325 to 385 μM, while PD patients presented values between 245 and 285 μM, suggesting that UA could be a potential marker for PD. Sensitive detection of DA has also been performed using electrolyte-gated field-effect transistors (EGFETs) with nanovesicles in a conducting polymer with immobilized human DA receptor D1 (Park et al., 2014). The authors reported a minimum detectable level of 1 nM for spiked DA in human serum, suggesting that this biosensor is suitable for PD diagnosis as DA reported values for PD are within the nM range. In a similar approach, Lee et al. developed a sensitive and reusable EGFET for DA detection using conductive polymer NPs coated with Pt particles (Lee et al., 2015), which act as catalysts for DA oxidation, enhancing signal detection, response time, and sensitivity. This sensor was able to detect DA in the fM concentration with minimal interference of AA or UA.

Although α-syn has been the most intensely studied and recognized biomarker for PD, its application in biosensing is very limited. A photoelectrochemical biosensor was developed by An et al. based on Au-doped TiO_2_ nanotube arrays for sensitive α-syn quantification with a detection limit of 34 pg/mL (An et al., 2010). Thrombin was been reported to induce apoptosis of dopaminergic neurons in rat *substancia nigra* (Choi et al., 2003) and microglia activation by inducing the expression of pro-inflammatory mediators TNFα, IL-1β, IL-6, and nitric oxide (Lee et al., 2005). Therefore, the detection and quantification of thrombin in the blood or CSF samples of PD patients could predict ongoing neuroinflammation while enabling disease diagnosis. An electrochemical biosensor for thrombin detection was developed by Bafrooei et al. using aptamers functionalized on a nanocomposite of multiwalled carbon nanotubes and TiO_2_ NPs (Heydari-Bafrooei et al., 2016). The aptasensor showed high specificity, sensitivity (in fM range) in blood, or CSF of PD patients.

The heterogeneous nature of MS, characterized by distinct patterns associated with the demyelination process, makes it highly improbable that a single diagnostic marker is capable of covering the full spectrum of MS subtypes (Lucchinetti et al., 2000). Lolli et al. developed a synthetic glycoprotein antigen probe, CSF114(Glc), for the specific recognition of autoantibodies present in the serum of MS patients (Lolli et al., 2005). The authors proved that the antibodies specific for CSF114(Glc) recognized myelin and oligodendrocyte autoantigens in human brain tissue. This knowledge enabled the development of a specific method for the identification of MS patients with antibody-mediated demyelination, a specific subset of MS patients. The same group later reported the development of a gold surface plasmon resonance (SPR) biosensor with covalently immobilized CSF114(Glc) for real-time MS diagnosis from serum (Real-Fernández et al., 2012). This SPR biosensor presented a mild sensitivity (36%) and elevated specificity (95%) relative to the identification of MS patients vs. healthy blood donors. Other than MS diagnosis, multiple autoantibody identification, and further clinical correlation could potentially be used to direct therapy and monitor its response.

## Conclusion

An increasing number of studies are uncovering the beneficial and detrimental roles of microglia in neurodegenerative disease onset and progression. Pro-inflammatory cytokines can be used in combination with classical biomarkers for neurodegenerative and neuroinflammatory disease diagnostics and monitoring of disease progression. Technologies for simultaneous detection and quantification of different biomarkers are rapidly developing and future devices are aimed at bringing valuable advantages, specifically related to lower sample volumes, detection time and limits, higher specificity and sensitivity. Decreasing the need for biological samples processing, while integrating biosensing platforms in portable lab-on-a-chip systems would, in turn, allow point-of-care use by semi-skilled operators toward real-time and *in situ* early diagnostics of neuroinflammatory and neurodegenerative diseases. Altogether, these advantages will surely bring great benefits for both academic and medical fields.

## Author contributions

All authors listed have made a substantial, direct and intellectual contribution to the work, and approved it for publication.

### Conflict of interest statement

The authors declare that the research was conducted in the absence of any commercial or financial relationships that could be construed as a potential conflict of interest.
